# Discovery of novel carbohydrate degrading enzymes from soda lakes through functional metagenomics

**DOI:** 10.3389/fmicb.2022.1059061

**Published:** 2022-12-07

**Authors:** Oliyad Jeilu, Addis Simachew, Erik Alexandersson, Eva Johansson, Amare Gessesse

**Affiliations:** ^1^Institute of Biotechnology, Addis Ababa University, Addis Ababa, Ethiopia; ^2^Department of Plant Breeding, Swedish University of Agricultural Sciences, Lomma, Sweden; ^3^Department of Plant Protection Biology, Swedish University of Agricultural Sciences, Lomma, Sweden; ^4^Department of Biological Sciences and Biotechnology, Botswana International University of Science and Technology, Palapye, Botswana

**Keywords:** extremophiles, glycoside hydrolases, lignocellulose biomass, halophiles, soda lakes, CAZymes, functional metagenomics

## Abstract

Extremophiles provide a one-of-a-kind source of enzymes with properties that allow them to endure the rigorous industrial conversion of lignocellulose biomass into fermentable sugars. However, the fact that most of these organisms fail to grow under typical culture conditions limits the accessibility to these enzymes. In this study, we employed a functional metagenomics approach to identify carbohydrate-degrading enzymes from Ethiopian soda lakes, which are extreme environments harboring a high microbial diversity. Out of 21,000 clones screened for the five carbohydrate hydrolyzing enzymes, 408 clones were found positive. Cellulase and amylase, gave high hit ratio of 1:75 and 1:280, respectively. A total of 378 genes involved in the degradation of complex carbohydrates were identified by combining high-throughput sequencing of 22 selected clones and bioinformatics analysis using a customized workflow. Around 41% of the annotated genes belonged to the Glycoside Hydrolases (GH). Multiple GHs were identified, indicating the potential to discover novel CAZymes useful for the enzymatic degradation of lignocellulose biomass from the Ethiopian soda Lakes. More than 73% of the annotated GH genes were linked to bacterial origins, with *Halomonas* as the most likely source. Biochemical characterization of the three enzymes from the selected clones (amylase, cellulase, and pectinase) showed that they are active in elevated temperatures, high pH, and high salt concentrations. These properties strongly indicate that the evaluated enzymes have the potential to be used for applications in various industrial processes, particularly in biorefinery for lignocellulose biomass conversion.

## Introduction

The most abundant bioresource on Earth, lignocellulosic biomass, has an annual global yield of up to 1.3 billion tons (Baruah et al., 2018; Zoghlami and Paës, 2019). Lignocellulosic biomass is mainly composed of polysaccharides (cellulose and hemicelluloses) and lignin (an aromatic polymer). Hydrolysis of the polysaccharide component of lignocellulosic biomass releases fermentable sugars (Anwar et al., 2014), which can produce renewable energy and chemicals (Abdel-Hamid et al., 2013). Such production can reduce dependence on fossil fuels, decrease greenhouse gas emissions, and mitigate climate change (Kabeyi and Olanrewaju, 2022).

Hydrolysis of lignocellulosic biomass can be done through acid or enzymatic hydrolysis. The acid hydrolysis effectively breaks down the polysaccharides into their monomeric sugars. However, it leads to the generation of inhibitors in subsequent fermentation (Oriez et al., 2019) and contributes to environmental pollution (Jönsson and Martín, 2016). Therefore, enzymatic hydrolysis, often carried out under relatively mild reaction conditions, offers an eco-friendly and efficient method for the hydrolysis of lignocellulosic biomass (Ellilä et al., 2019). In practice, the complete breakdown of lignocellulosic polysaccharides to their monomeric components requires the synergistic action of multiple enzymes, known as Carbohydrate-Active enZymes (CAZymes) (Bredon et al., 2018; Liew et al., 2022).

Industrial processes for lignocellulosic biomass degradation has to operate at high temperatures to increase substrate accessibility. In addition, alkali pretreatment is used to enhance the internal surface area of hemicelluloses while removing acetyl groups and uronic acids (Baruah et al., 2018; Lu et al., 2021; Sun et al., 2021; Verma, 2021). Therefore, enzymes that have activity and stability in an alkaline pH range and/or at high temperatures have a potential for hydrolysis of lignocellulosic biomass (Ariaeenejad et al., 2020; Mamo, 2020). However, most known cellulases and hemicellulases are obtained from mesophilic organisms and have their optimum activity and stability around ambient temperature and in a neutral pH range (Collins et al., 2005; Ben Hmad and Gargouri, 2017). To date, relatively few alkaline active and thermostable cellulases and hemicellulases have been reported (Shuddhodana et al., 2018; Verma, 2021). Furthermore, a significant hurdle to the large-scale conversion of lignocellulose to biofuels is the scarcity of low-cost enzymes capable of effectively depolymerizing biomass (Kern et al., 2013). Thus, there is a high need to search for novel enzymes not only to enhance bioconversion but also to in order to make the process more environmentally friendly and cost-effective (Fongaro et al., 2020; Verma and Satyanarayana, 2020; Verma, 2021). One of the best strategies to obtain such enzymes is to search for lignocellulose polysaccharide degrading enzymes from extreme environments such as soda lakes (Berini et al., 2017a; Cabrera and Blamey, 2018).

Soda lakes are unique poly-extreme environments, mainly characterized by high alkalinity and salinity (Schagerl, 2016) and harbor unique and diverse microbial communities (Lanzén et al., 2013; Sorokin et al., 2015; Grant and Jones, 2016). These microbial communities from soda lakes provide a one-of-a-kind source of enzymes (Antony et al., 2013) with properties that allow them to endure the rigorous industrial conversion of lignocellulose biomass into fermentable sugars. Cellulases, hemicellulases, and other carbohydrate polymer degrading enzymes produced by microorganisms from these habitats are expected to be active and stable under extreme conditions prevalent in the soda lakes.

Although most of the known industrial enzymes are obtained from microorganisms through pure culture isolation and screening (Martin and Vandenbol, 2016), recent estimates show that more than 99% of microorganisms in the environment are uncultivable through conventional microbiological methods (Berini et al., 2017a). Thus, in recent years the advent of metagenomics has provided a powerful tool to access the genetic and metabolic diversity of microorganisms in any environment, bypassing the limitations of the current culture-dependent approaches (Berini et al., 2017a; Ngara and Zhang, 2018; Pabbathi et al., 2021). Functional metagenomics is a method that clones the environmental DNA into a suitable vector to create a library and which is then transformed into a host, such as *E. coli*. This library is then screened for various enzymatic activities (Simon and Daniel, 2011; Coughlan et al., 2015; Lam et al., 2015; Sysoev et al., 2021). Through functional metagenomics, novel biocatalysts, including lipases (Privé et al., 2015), cellulases (Voget et al., 2006), amylases (Vester et al., 2015), and chitinases (Berini et al., 2017b) have been discovered.

Whereas CAZymes from ruminants (Wang et al., 2013, 2019; Shen et al., 2020), lignocellulosic biomass wastes (Montella et al., 2017), and soil (López-Mondéjar et al., 2020), have already been thoroughly explored, investigations of CAZymes produced by microbial communities of soda lakes remain scarce. Thus, the main objective of this study was to search for CAZymes from the Ethiopian Rift Valley soda lakes using functional metagenomics.

## Materials and methods

### Sampling site and sample collection

Water and sediment samples were collected from three soda lakes in the East African Rift Valley: Lakes Abijata (7°37′0″N, 38°36′0″E), Chitu (7°24′14″N, 38°25′15″E), and Shala (7°25′29″N, 38°36′57″E), using sterile Niskin bottles (Ocean Scientific International Ltd., Hampshire, United Kingdom) and polyethylene bags, respectively. The water samples were filtered within 24 h of sample collection, using a polycarbonate filter membrane (0.22 μm pore size, 47 mm diameter; GE, IL, United States) to harvest the biomass for metagenomic DNA extraction.

### Isolation of high molecular weight DNA

Metagenomic DNA was extracted from water and sediment samples according to Øvreås et al. (2003) and Verma and Satyanarayana (2011), respectively, with some modifications described below. Briefly, about 250 μl solution of 1 mg/ml Lysozyme and 0.5 mg/ml RNase (Thermo Scientific, Massachusetts, United States) was added to the microbial biomass on the polycarbonate filter membrane and incubated for 15 min at 37°C. The filter was then treated with 10 μl of 1 mg/ml Proteinase K (Thermo Scientific, Massachusetts, United States) and incubated for 15 min at 37°C. Afterwards, 250 μl of pre-heated 10% sodium dodecyl sulfate (SDS; *w*/*v*) was added and incubated for 15 min at 55°C. About 80 μl of 5 M NaCl and 100 μl of 1% cetyltrimethylammonium bromide (CTAB; *w*/*v*) were added and incubated for 10 min at 65°C; followed by the addition of 750 μl chloroform/isoamyl alcohol (24:1). The mixture was centrifuged at 12,000 × *g* for 15 min, and then a 0.6 volume of isopropanol was added to precipitate the aqueous layer. Finally, the mixture was centrifuged at 12000 *g* for 15 min, and the pellet was washed with 70% (*v*/*v*) ethanol, dried at room temperature, and dissolved in 10 mM TE buffer (pH 8.0). To extract DNA from sediment samples, about 10 g of sediment was suspended in 13.5 ml of extraction buffer (1%CTAB, *w*/*v*), 100 mM Tris (pH 8.0), 100 mM NaH_2_PO_4_ (pH 8.00), 100 mM EDTA, 1.5 M NaCl). Then, 50 μl of 10 mg/ml proteinase K (Thermo Scientific, Massachusetts, United States) was added, and the mixture was incubated at 37°C for 30 min. After that, 1.5 ml of 20% SDS was added and incubated for 2 h at 65°C. The samples were centrifuged at 4,000 × *g* for 20 min at room temperature to separate the sediment remnants from the cell lysates. The cell lysates were mixed with an equal proportion of phenol, chloroform, and isoamyl alcohol (25:24:1) and were centrifuged at 16,000 × *g* for 5 min. Then, the DNA in the aqueous layer was precipitated by adding 0.6 volumes of isopropanol and recovered by centrifuging at 16,000 × *g* for 10 min. Finally, the pellet was washed with 70% (*v*/*v*) ethanol, dried by air, and dissolved in 10 mM TE buffer (pH 8.0). The quantification and quality control of extracted DNA were performed using a Nanodrop (Thermo Scientific, Massachusetts, United States) and gel electrophoresis with 0.8% agarose gel (Thermo Scientific, Massachusetts, United States).

### Construction of the metagenomic library

The metagenomic libraries were constructed by the CopyControl^™^ Fosmid Library Production Kit (Epicentre, Madison, United States) according to the manufacturer’s protocol. Briefly, the high molecular weight metagenomic DNA was sheared to the appropriate size by passing it through a 200-μl small-bore pipette tip a few times and then end-repaired using the CopyControl blunt-end repair kit (Epicentre, Madison, United States). The end-repaired DNA was loaded onto a 1% low-melting-point agarose gel for 16–20 h at 35 voltage in 1× TAE buffer. The DNA fragments that were run parallel to the 40 kb Fosmid control DNA were excised from the gel without UV light. The DNA from the excised gel was recovered using the GELase protocol (Epicentre, Madison, United States), followed by a conventional phenol:chloroform:isoamyl alcohol (24:24:1) extraction to remove the enzymes, and the DNA was precipitated using isopropanol. The resultant DNA pellet was resuspended in 10 mM TE (pH 8.0), and the yield and size of the DNA were validated by running an aliquot down the gel. The end-repaired DNA was ligated to the CopyControl pCC1FOS (Epicentre, Madison, United States) vector at a 10:1 vector-to-insert DNA ratio. The ligated DNA was then packaged into the MaxPlax lambda phage heads (Epicentre, Madison, United States). The lambda phage packaged DNA was then infected into *E. coli* cells (EPI100-T1R Plating Strain, Epicentre, Madison, United States), mixed 2–3 times by inverting the tubes and incubated for 30 min at room temperature. The infected cells were then plated on prewarmed Luria-Bertani (LB) agar plates (supplemented with 12.5 μg/ml Chloramphenicol) and incubated at 37°C for 16–20 h. After the quality control, single colonies were manually transferred to individual wells on a 384-well microtiter plate (Thermofisher, Madison, United States) with 60 μl LB medium supplemented with 12.5 μg/ml chloramphenicol and 20% glycerol. The library was duplicated from each master plate and kept at –80°C for further screening.

### Functional screening of specific genes in the fosmid library

The library was replicated to LB agar plates supplemented with soluble starch (1%; Sigma-Aldrich, Missouri, United States), carboxyl methylcellulose (1%; CMC, Sigma-Aldrich, Missouri, United States), pectin (1%; Sigma-Aldrich, Missouri, United States), and xylan (1%; Sigma-Aldrich, Missouri, United States), to detect amylase, cellulase, pectinase, and xylanase activities, respectively. Whereas to detect β-glucosidase activity, LB agar plates were supplemented with esculin hydrate (0.1%; Sigma-Aldrich, Missouri, United States) and ferric ammonium citrate (0.25%; Sigma-Aldrich, Missouri, United States). In addition, LB agar plates were supplemented with chloramphenicol (12.5 μg/ml) and Autoinduction solution (0.2%; Epicentre, Madison, United States). Then, the plates were incubated at 37°C for 2–7 days. For the identification of cellulase and xylanase-positive clones, the method proposed by Teather and Wood (1982) was used, where the colonies were washed off the agar plates with ddH_2_O to permit a homogeneous penetration of the staining dye into the medium. Thereafter, the agar plates were stained with 0.2% Congo red solution for 30 min. After the solution was poured off, the agar plates were de-stained up to 3 times for 30 min with 1 M NaCl. Positive clones were detected by forming a yellow halo against a red background. The β-glucosidase activity was detected according to Eberhart et al. (1964) method, where clones exhibiting a black halo around their colonies were selected as positive for β-glucosidase activities. Amylase activity was detected by staining the plates with KI/I_2_ solution. A colorless halo surrounded positive colonies on a dark purple background (Sharma et al., 2010).

### Fosmid DNA extraction and sequencing

A total of 22 fosmid clones with positive enzyme activities were selected, cultured and induced to a high copy number using the 500 × Copy Control Fosmid Autoinduction Solution (Epicentre, Madison, United States) at 37°C overnight (16–20 h) with 12.5 μg/ml chloramphenicol and vigorous shaking (225–250 rpm). Fosmid DNA was extracted using a GenElute Plasmid Miniprep Kit (Sigma-Aldrich, Missouri, United States) according to the manufacturer’s protocol. The quality of extracted DNA was checked using a Nanodrop and sent to BGI Genomics for whole-genome sequencing. The *E. coli* whole genome resequencing was performed using the paired-end 150 bp method using the DNBSEQ^™^ platform.

### Bioinformatics analysis

#### De-novo assembly

An in-house workflow was developed to analyze the whole genome sequences of the fosmids.^1^ Briefly, whole genome sequencing (WGS) raw reads Quality Check (QC) was performed with the FastQC tool (Andrews, 2010), and multiple samples were processed with MultiQC (Ewels et al., 2016). All reads from independent 22 fosmid clones were mapped to the *E. coli* reference genome (EPI100-T1R Plating Strain, Epicenter Biotechnologies) using Bowtie2 (Langmead and Salzberg, 2013) and reads were sorted with SAMtools (Danecek et al., 2021). Reads that did not map to the *E. coli* genome were further used for de-novo assembly with SPAdes v3.15.3 and metaplasmidspades (Bankevich et al., 2012). Further, the CopyControl pCC1FOS (Epicenter Biotechnologies) vector backbone sequences were removed from the assembled contigs. NCBI-Blast-v2.9.0^+^ and Bedtools-v2.27.1 were used for processing assemblies.

#### Gene prediction, taxonomy, and functional annotation

Functional annotation of the assembled fosmid contigs were performed using RASTtk (Brettin et al., 2015) webserver, and cross-validation of predicted coding DNA sequence (CDS) using TransDecoder-v5.5.0.^2^ Then, carbohydrate-active enzymes (Cantarel et al., 2009) annotation was conducted using hmmscan (Version 3.3 b2) against CAZy database (version 2021). Kraken2 (version 2.0.8) (Wood et al., 2019) was applied for taxonomic sequence classification using the Minikraken2_v1_8GB database. Finally, KronaTools v2.7.1 (Ondov et al., 2011) was used to visualize taxonomies.

#### Preparation of cell culture for enzymatic assay

Among the enzymes screened, amylase, cellulase, and pectinase were selected based on their potential application in complex carbohydrate degradation and their enzymatic activity studied. These positive clones were preliminarily assessed based on their clearing zone diameter, and the top four clones in each enzyme were chosen for further characterization. Positive clones (pectinase, cellulase, and amylase) were inoculated in LB media (50 ml) containing chloramphenicol (12.5 μg/ml) and Autoinduction solution (0.2%), supplemented with 1% of their respective substrates (Pectin/carboxyl methylcellulose/soluble starch). Then, the inoculated media were incubated at 37°C, 200 rpm for 3–5 days, and after incubation, centrifuged at 5,000 × *g* and 4°C for 30 min. The supernatant (crude cell-free extract) was used as a crude enzyme cti fraction for further analysis.

### Enzyme assays

Enzyme activity was measured following the dinitrosalicylic acid (DNS) method (Miller, 1959). The standard assay mixture contains 100 μl of the enzyme or crude cell-free extract and 250 μl of the substrate (1% in a final volume of 0.5 ml), and 150 μl McIlvaine buffer. Carboxymethyl cellulose (CMC), pectin, and soluble starch were used as substrates for cellulase, pectinase, and amylase, respectively. The mixture was incubated at 37°C for 15 min, then 750 μl DNS reagent was added, and the samples were boiled at 100°C for 15 min. After cooling down on the ice, the samples were centrifuged at 16,000 × *g* for 2 min to precipitate falling proteins. The samples were transferred to cuvettes, and absorbance was measured at 540 nm (Thermo Scientific). The pH range of the enzyme was determined by measuring standard assay activity between pH 7.0 and 10.5 using 50 mM of appropriate buffers. The temperature range of the enzyme activity was determined by assaying at temperatures between 30 and 75°C. The effect of salt on enzyme activities was investigated by incorporating 0-3M of NaCl into the reaction mixture.

## Results

### Construction and functional screening of metagenomic libraries

Metagenomic libraries with more than 21,000 clones were generated from three Ethiopian soda lakes (Table 1). At the time of sampling, the salinity of the lakes ranged from 3% (Lake Shala) to 15% (Lake Abijata) and pH from 9.3 (Lake Shala) to 10.0 (Lake Chitu). The number of clones picked from each sample site ranges from 1,440 (Lake Abijata) to 10,368 (Lake Shala; Table 1).

**Table 1:** Physical parameters of the studied soda lakes of Ethiopia with the number of metagenomics libraries constructed.

Sample site	pH	Salinity (%)	Number of clones
Abijata	9.5	15	1,440
Chitu	10	6	9,216
Shala	9.3	3	10,368
	Total		21,024

**Figure 1:**
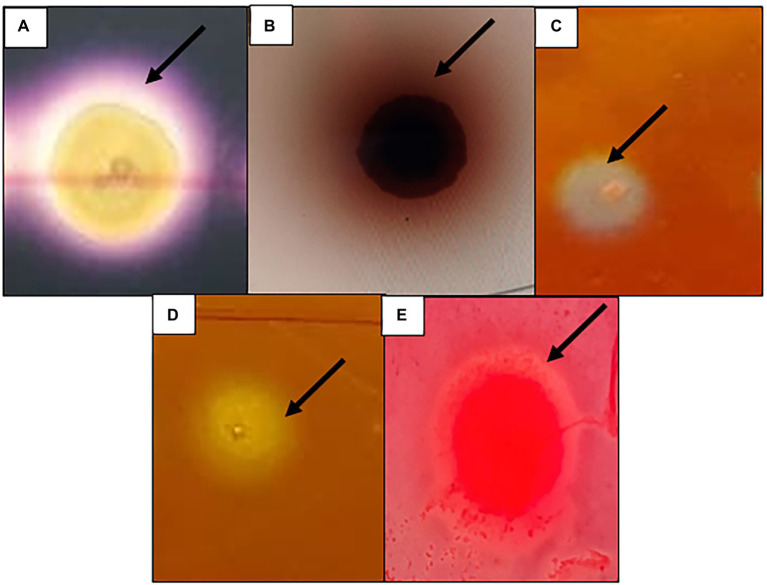
Functional screening of the metagenomics library constructed from Ethiopian soda lakes showing positive for **(A)** Amylase, **(B)** β-glucosidase, **(C)** Cellulase, **(D)** Pectinase, **(E)** Xylanase.

Among all the clones screened (> 21,000) for the five hydrolytic enzymes, a total of 408 clones were positive for at least one of the hydrolytic enzymes tested (Figure 1). A relatively high number of clones (281 clones, 1.33% hit ratio) were positive for cellulase, followed by amylase (75 clones, 0.36% hit ratio), β-glucosidase (20 clones, 0.1% hit ratio), pectinase (15 clones, 0.07% hit ratio) and 12 positives for xylanase (12 clones, 0.06% hit ratio; Table 2).

**Table 2:** Hit ratios of the screened hydrolytic enzymes.

Enzyme	Hit ratio
Amylase	1:280
B-glucosidase	1:1051
Cellulase	1: 75
Pectinase	1:1402
Xylanase	1: 1752

### Analysis of fosmid insert sequences and detection of carbohydrate-active enzymes

A total of 22 clones that were positive for the enzymes mentioned above were selected and subjected to sequencing. After bioinformatics sequence analysis and assembly, the insert sizes were between 30,230 and 44,461 base pairs. Each library contained clones with average size inserts of approximately 35 kb, yielding approximately 0.74 GB of the total cloned genomic DNA per library. A total of 1,233 open reading frames (ORFs) were predicted from the fosmid clones (Table 3). Then, the carbohydrate-active enzyme (CAZy) database was used to annotate the predicted ORFs, resulting in 378 encoding genes with potential functions predicted (Supplementary Table 1). All the detected genes coding for CAZymes were further assigned to six functional classes: 10 Auxiliary Activities (AAs), 61 Carbohydrate-Binding Modules (CBMs), 24 Carbohydrate Esterases (CEs), 156 Glycoside Hydrolases (GHs), 118 Glycosyl transferases (GTs), and 9 Polysaccharide Lysases (PLs; Table 3).

**Table 3:** The DNA Insert size of each fosmid in base pairs, the number of ORFs, and detected CAZyme classes.

Fosmid clones	Contig size (bp)	GC (%)	ORFs	AAs	CBMs	CEs	GHs	GTs	PL	Total CAZYmes
F1	39,939	59.81	77	0	0	5	5	0	0	10
F2	33,919	44.52	34	0	0	0	10	17	0	28
F3	30,230	53.28	34	0	1	0	0	0	0	1
F4	43,585	63.87	75	0	1	1	2	5	0	9
F6	31,316	52.00	36	2	15	4	27	11	1	60
F8	36,493	47.93	39	0	3	1	11	9	1	25
F9	36,313	57.36	67	1	2	1	7	4	2	17
F10	44,461	64.38	79	0	1	1	5	2	0	10
F11	31,461	64.19	72	0	0	0	1	0	0	1
F13	37,445	65.37	83	0	2	1	6	4	0	13
F14	36,784	60.12	68	0	1	2	5	6	0	14
F15	42,189	65.68	87	1	2	0	4	6	3	16
F16	33,691	49.63	39	0	1	1	4	1	0	7
F17	35,026	58.60	58	0	5	2	7	4	0	18
F18	35,903	56.98	68	0	3	0	8	4	1	16
F19	35,357	51.12	44	0	0	1	9	7	0	17
F20	31,419	65.25	72	0	3	0	3	7	0	13
F21	33,565	49.65	39	0	1	1	4	7	0	13
F22	35,225	52.03	40	1	3	4	14	6	0	28
F23	36,297	47.81	28	1	15	1	18	6	1	42
F24	38,076	53.27	40	0	0	0	3	9	0	16
F25	30,893	52.05	54	0	0	1	2	2	0	5
Total			1,233	10	61	27	156	117	9	378

ORFs, open reading frames; AAs, auxiliary activities; CBMs, carbohydrate-binding modules; CEs, carbohydrate esterases; GHs, glycoside hydrolases; GTs, Glycosyl transferases; PLs, polysaccharide lyases

### Glycoside hydrolase enzymes

**Figure 2:**
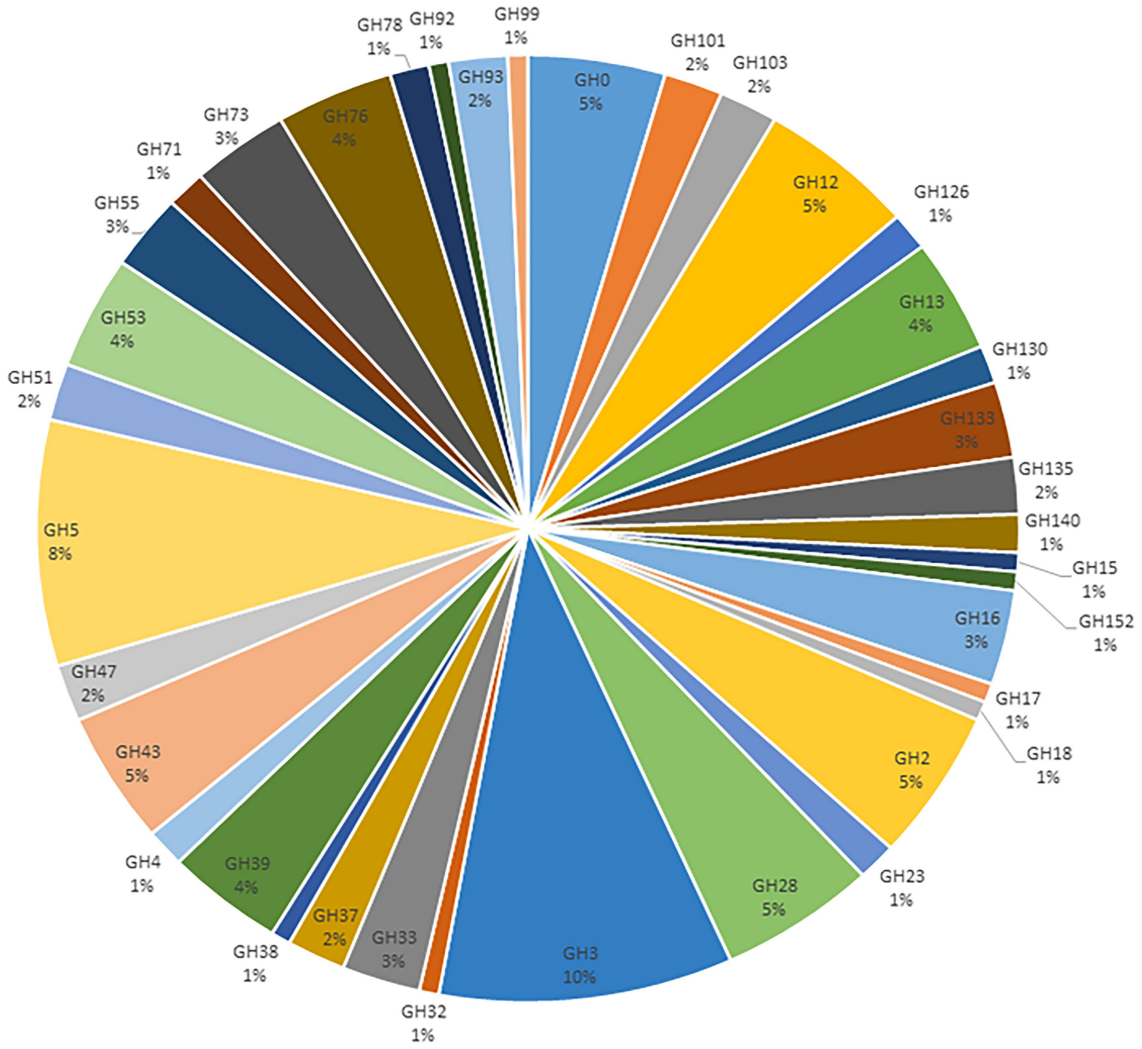
Classification of the annotated GH families from the metagenomics libraries of Ethiopian soda lakes.

GHs were the most abundant class, representing 40% of the identified carbohydrate-degrading enzymes (Table 4; Supplementary Table 1). These GHs were categorized into 38 GH families. The most abundant GH families were GH2, GH3, GH5, GH12, GH13, and GH28. About 10% of the GH families belonged to the GH3 family, which encodes β-glucosidase (EC 3.2.1.21), and N-acetyl-β-D-glucosaminidases (EC 3.2.1.30). The GH5 family representing Cellulase (EC 3.2.1.4) were the second most abundant GH family, representing 8% of the total families. About 5% of the GH families were found to belong to the GH28, a family that contains pectinase polygalacturonase enzymes. Starch degrading enzymes such as alpha-amylases (GH13) were also abundant (4%; Figure 2).

**Table 4:** Carbohydrate degrading enzymes detected in the fosmid clones (the complete list in Supplementary Table).

GH family	Fosmid	Hit accession	Identity (%)	Predicted enzyme
GH3	F17	ATH78710.1	96.8	β-glucosidase
GH103	F24	AVI63997.1	86.4	Peptidoglycan lytic transglycosylase
	F11	AXY42412.1	85.5	
GH13	F15	AHO18837.1	69.0	α-amylase
GH73	F19	AZU02762.1	51.9	Lysozyme
GH47	F13	CBX90714.1	47.8	α-mannosidase
GH2	F15	ACR62057.1	47.5	β-galactosidase
GH3	F9	SQK97389.1	46.3	β-glucosidase
GH13	F13	QTJ41571.1	46.0	α-amylase
GH78	4F4	ACT97502.1	43.0	α-L-rhamnosidase
GH43	F14	BBI53471.1	42.0	β-xylosidase
GH3	F22	SQK97389.1	40.7	β-glucosidase
	F6	SQK97389.1	40.5	
GH5	F8	QXC62151.1	40.4	Cellulase
GH38	F1	QJS43890.1	40.1	α-mannosidase
GH140	F22	QJW98933.1	39.6	β-1,2-apiosidase
	F6			
GH53 GH53	F22	QKX57828.1	39.5	Endo-β-1,4-galactanase
	F6			
GH55	F25	ATI54262.1	39.4	Exo-β-1,3-glucanase
GH5	F25	QXC62151.1	39.4	Cellulase
GH103	F20	BCX45957.1	38.1	Peptidoglycan lytic transglycosylase
GH92	F17	AGB27560.1	38.1	α-1,2-mannosidase
GH51	F17	QKX56076.1	37.9	Endoglucanase
GH5	F10	QXC62151.1	37.6	Cellulase
GH51	F19	QKX56076.1	37.4	Endoglucanase
GH55	F14	AOW25396.1	36.7	Exo-β-1,3-glucanase
GH5	F23	QXC62151.1	36.0	Cellulase
GH99	F13	CAZ29389.1	36.0	α-1,2-mannosidase
GH3	F10	SQK97389.1	35.8	β-glucosidase
GH37	F19	AWO98658.1	35.4	α-trehalase
GH5	F9	QXC62151.1	35.4	Cellulase
GH23	F9	APT58734.1	35.4	Lysozyme
GH51	F8	QKX56076.1	35.0	Endoglucanase

EC, Enzyme commission number.

### Tracking the microbial sources of the annotated GH genes

The microbial source of the annotated GH genes were predicted, and more than 73% of the genes aligned to bacterial sources. However, there were no organism hits for about 27% of the annotated GH genes. Most of the bacterial genes at the class level belonged to the *Gammaproteobacterial* and at the genera level to *Halomonas* (Supplementary Figure 1).

### Expression and characterization of selected enzymes

#### Cellulase

**Figure 3:**
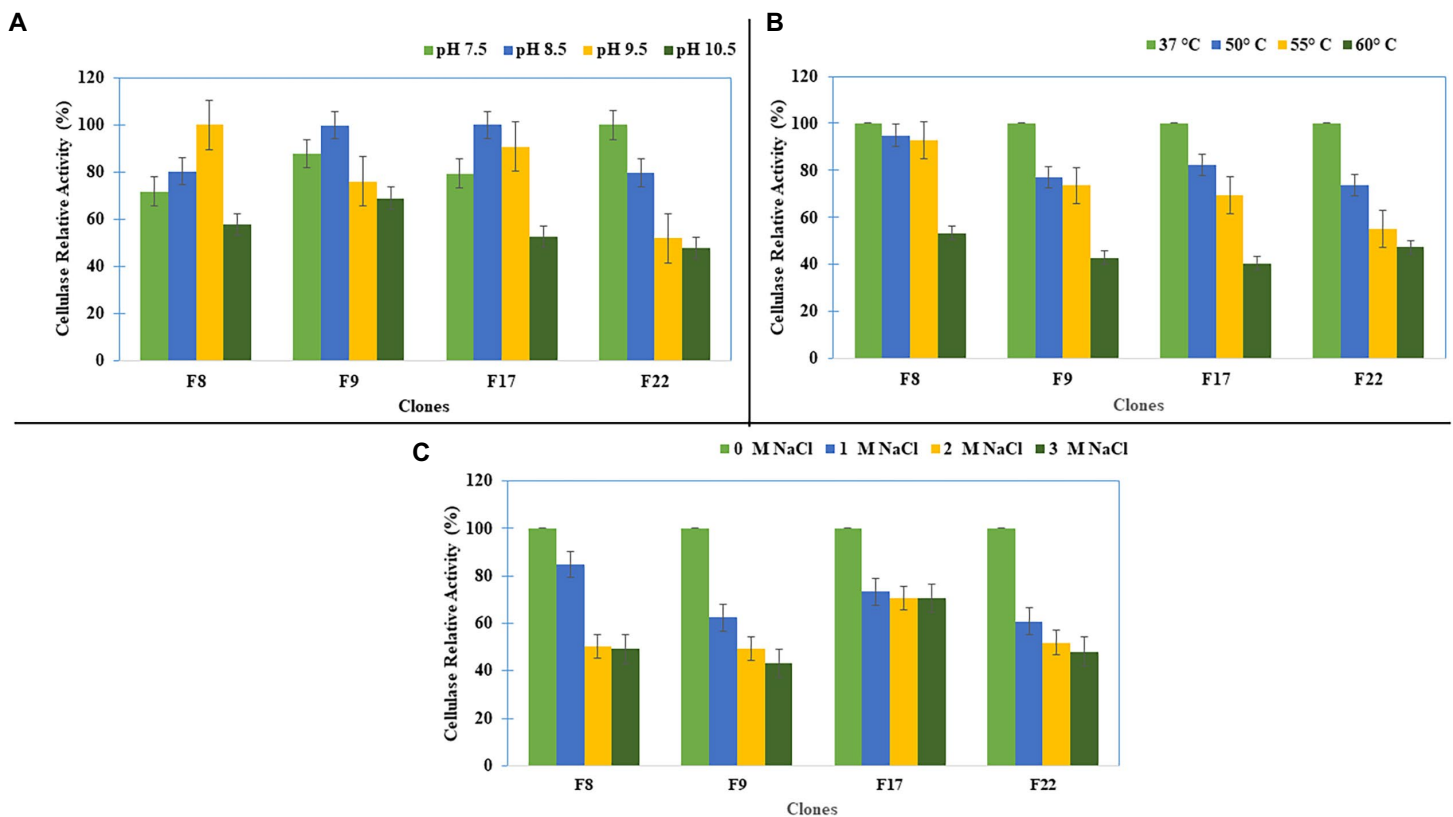
Cellulase Activity characterization of the clones for **(A)** pH, **(B)** Temperature, and **(C)** Salt concentration.

Further studies on the effect of pH, temperature, and salt concentration on the cellulase activities of the selected clones (F8, F9, F17, and F22) showed that the clone F8 had its optimum cellulase activity at pH 9.5, whereas clones F17 and F9 showed the optimum activity at pH 8.5 (Figure 3A). All clones showed their optimum cellulase activity at 37°C (Figure 3B). The optimum cellulase activities for all clones were at 0M NaCl, while the clone F17 maintained 75% of the cellulase activity up to 3 M salt concentration (Figure 3C).

#### Amylase

**Figure 4:**
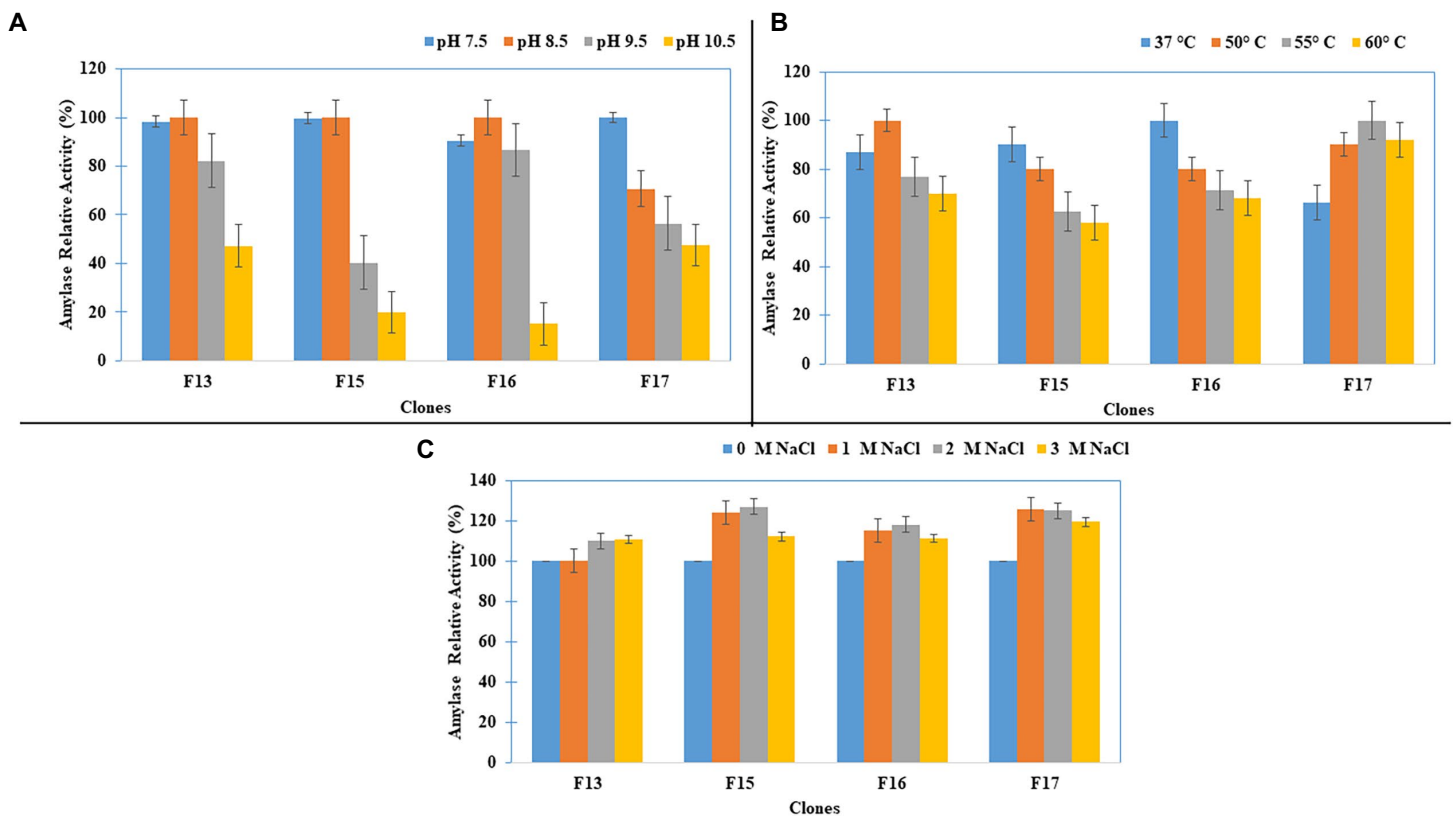
Amylase Activity characterization of the clones for **(A)** pH, **(B)** Temperature, and **(C)** Salt concentration.

Studies on the effect of pH, temperature and salinity on the amylase activity of four screened fosmid clones (F13, F15, F17, and F22) showed that all four clones, except for F22, had the maximum amylase activity at pH 8.5 (Figure 4A), and that the clones F17, F13, and F15 showed the optimum amylase activity at 55, 50, and 45°C, respectively (Figure 4B). Furthermore, these amylase-positive clones showed increasing relative amylase activity with increased salinity (Figure 4C).

#### Pectinase

**Figure 5:**
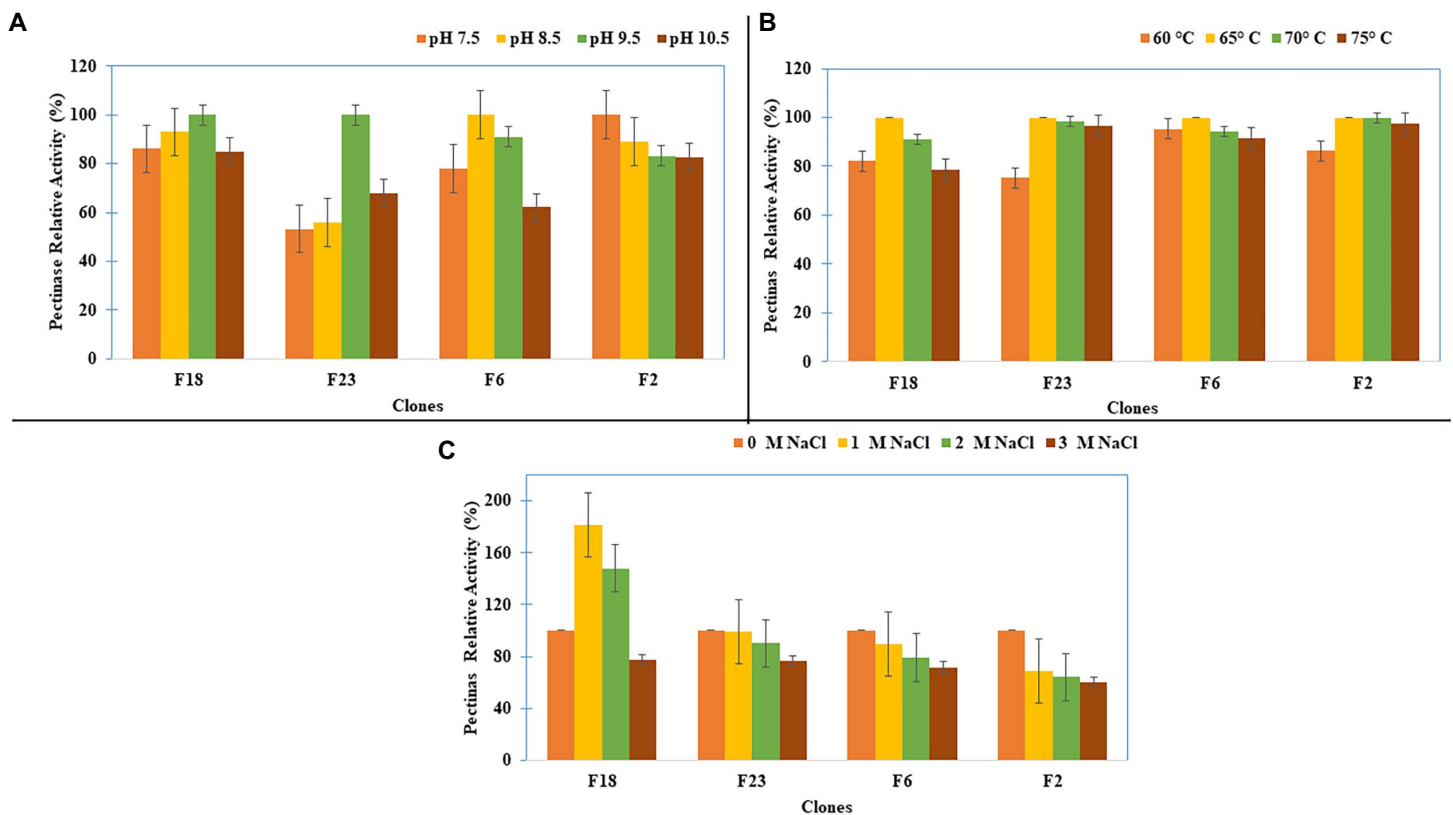
Pectinase Activity characterization of the clones for **(A)** pH, **(B)** Temperature, and **(C)** Salt concentration.

Studies on the pectinase activity of four fosmid clones (F2, F6, F18, and F23) showed that the clones F18 and F23 had their optimum pectinase activity at pH 9.5 (Figure 5A) and that all four clones had optimum pectinase activity at 65°C (Figure 5B). When different salt concentrations were added, clone F18 showed enhanced pectinase activity, while others retained more than 75% of the pectinase activity up to 3M salt concentrations (Figure 5C).

## Discussion

Despite the potential of soda lakes as sources of unique extremozymes, only a few enzymes have so far been identified from these ecosystems. Previously, carbohydrate-degrading enzymes, i.e., xylanase (Gessesse and Mamo, 1998), amylases (Martins et al., 2001), and cellulase (Minig et al., 2009), have been discovered from Ethiopian soda lakes. However, these studies have been based on conventional culture-dependent approaches, in which most microorganisms are unable to grow, thereby limiting the identification of novel enzymes (Culligan et al., 2014). In this study of the Ethiopian soda lakes, we used a functional metagenomics approach to discover carbohydrate-degrading enzymes (CAZymes) to circumvent this limitation.

The DNA extracted from the Ethiopian soda lakes was used to construct metagenomic libraries, which generated clones that produced carbohydrate-degrading enzymes. The hit ratios obtained for the hydrolytic enzymes, especially cellulase and amylase, were high (1:75 and 1:280, respectively) compared to many previous reports in other environments (Ferrer et al., 2012; Nguyen et al., 2012; Liu et al., 2015; Wang et al., 2015; Maruthamuthu et al., 2016). The functional screening approach has previously been reported to be superior in finding genes producing functional products, which increases the potential to uncover entirely new classes of enzymes that lack homologies to previously identified sequences (Culligan et al., 2014; Berini et al., 2017a). The high hit ratios of this study might largely be attributed to the source of the metagenome, i.e., the abundance and diversity of microorganisms producing the different carbohydrate-degrading enzymes in the Ethiopian soda lakes. The Ethiopian soda lakes are characterized by high primary production due to the presence of a dense population of haloalkaliphilic cyanobacteria (Wood et al., 1984; Grant and Sorokin, 2011), which in turn support a diverse group of heterotrophic prokaryotes (Lanzén et al., 2013; Grant and Jones, 2016). Since some of the heterotrophic microorganisms are involved in nutrient recycling released by dead cells, many of them are expected to produce different hydrolytic enzymes, including those involved in the hydrolysis of complex carbohydrates, such as cellulose, hemicellulose, pectin and starch (Jones and Grant, 1999; Sorokin and Kuenen, 2005; Sorokin et al., 2014). The presence of a diverse group of heterotrophs producing the above enzymes might, in turn, lead to an observed high hit ratio.

The complete breakdown of lignocellulosic polymers requires the synergistic action of multiple CAZymes (Bredon et al., 2018; Liew et al., 2022), i.e., mainly consisting of the Glycoside Hydrolases (GHs) families such as cellulase, xylanase, hemicellulase, and pectinase (Rastogi and Shrivastava, 2020; Shrivastava, 2020). About 41% of the CAZymes database annotated genes obtained from the Ethiopian soda lakes have been shown to belong to GHs. GHs are the best-described families of carbohydrate-degrading enzymes due to their high prevalence and broad distribution across genomes (Berlemont and Martiny, 2015; Stewart et al., 2018; Wang et al., 2019). Microorganisms in soda lakes degrade complex polysaccharides, releasing short metabolizable oligosaccharides into the lake environment. Detecting multiple GH enzymes, including GH5, GH3, GH13, GH43, and GH28, indicates the relevance of these enzymes to the ecosystems of the soda lakes and their carbon cycle. The short metabolizable oligosaccharides produced are fed into the core carbohydrate metabolic pathways, providing energy and precursor metabolites for other pathways (Salam, 2018).

Among the components of lignocellulosic biomass, cellulose is the most prevalent biopolymer on the planet (Lakhundi et al., 2015). In nature, microorganisms involved in nutrient recycling in the ecosystem, including soda lakes, enzymatically hydrolyze cellulose to its monomeric unit and use it as a nutrient for growth. Different cellulase enzymes are required to achieve complete hydrolysis of cellulose, the main ones being endoglucanases and β-glucosidases (Tiwari et al., 2016). In this study, about 10 and 8% of the GHs families belonged to GH3 and GH5, respectively. The GH3 and GH5 families possess β-glucosidase (EC 3.2.1.21) and endo-1,4-glucanase (EC 3.2.1.4) activities, respectively. The GH5 enzymes are involved in the hydrolysis of cellulose into cellobiose (Wang et al., 2019), while GH3 enzymes break down cellobiose into glucose (Agirre et al., 2016). Members of the GH5 superfamily are extensively dispersed across archaea, bacteria, and eukaryotes, and various enzyme functions related to biomass conversion have been discovered in this superfamily (Mohammadi et al., 2022). In cellulose biomass degradation, β-glucosidase (GH3) activity is regarded as the rate-limiting factor, reducing cellobiose inhibition on endoglucanases and permitting more effective cellulolytic enzyme action (Tiwari et al., 2017; Zhang et al., 2017).

Among the hemicellulase enzymes, GH43 was the most abundant family in the present study. Previous studies have reported the enzyme activities of GH43 to be β-xylosidase (EC 3.2.1.37), xylanase (EC 3.2.1.8) and exo-β-1,3-galactanase (EC 3.2.1.145; (Shrivastava, 2020), which are enzymes contributing to the degradation of xylan (the main component of hemicellulose) to xylose monomers (Houfani et al., 2021). One major challenge in producing biofuels from lignocellulosic biomass by enzymatic hydrolysis is that hemicellulose and lignin create a protective barrier surrounding the cellulose. Furthermore, the crystalline form of cellulose renders it insoluble and resistant to enzyme degradation (Zoghlami and Paës, 2019; Reichart et al., 2021). Thus, the removal of hemicellulose may result in increased access of cellulase to cellulose (Peng et al., 2015). Previous studies have shown that for hemicellulose degradation, the numerous GHs enzymes operating at various levels of the hemicellulolytic matrix are needed (Horn et al., 2012; Andlar et al., 2018). In this context, the breakdown of hemicelluloses by a monofunctional hemicellulase enzyme is uneconomical. However, the multifunctional hemicellulase activity of GH43 (primarily in the xylan component) can minimize enzyme costs and is essential for the complete hydrolysis of lignocellulosic biomass (Limsakul et al., 2021).

About 5% of GHs belonged to the family of GH28. The GH28 families have been shown to encode polygalacturonase, which acts on the 1,4-glycosidic bond, and plays a crucial part in pectin digestion (Zhao et al., 2014). The architectural characteristics of cell walls, which have been represented as a cellulose-hemicellulose network embedded in a pectin matrix, imply that pectins may hide cellulose and/or hemicellulose, preventing degradative enzymes from attacking them (Xiao and Anderson, 2013). Thus, pectin degradation is needed for the complete degradation of lignocellulose biomass. A recent study has shown that some haloalkaliphile *Bacteroidota* and *Clostridia* from soda lakes utilize pectin as a substrate (Sorokin et al., 2014).

Furthermore, approximately 4% of the GHs in this investigation belonged to GH13, which encodes amylases. The Ethiopian soda lakes are known to have one of the highest primary productivity of any natural habitat due to the thick biomass of cyanobacteria (Faris, 2017), which frequently accumulate starch (10–50%) as part of their biomass (Möllers et al., 2014). Therefore, these heterotrophic microorganisms are known to produce abundant hydrolytic enzymes, such as amylases, to allow the breakdown of starch (Sorokin et al., 2014).

Soda lakes are a unique source of microorganisms that thrive in high alkalinity and salinity (Sorokin et al., 2014). These microorganisms produce either haloalkaliphilic or haloalkali-tolerant hydrolytic enzymes. The haloalkaliphilic enzymes usually show optimal activity in high alkaline and saline ranges. On the other hand, the haloalkali-tolerant enzymes may work best at a neutral pH and without salt. Still, these enzymes are active and stable in the high alkalinity and salinity range (DasSarma and DasSarma, 2015). The cellulases obtained in this study were active and stable in alkaline conditions, high temperatures and salt concentrations, indicating these enzymes are haloalkali-tolerant. This is contrary to the majority of reported cellulases, which demonstrated limited activity in these multi-extreme circumstances, such as in the presence of alkaline and salinity, and just a few alkalophilic and halotolerant cellulases have been identified (Zhao et al., 2012; Garg et al., 2016). The conversion of lignocellulosic biomass usually occurs at high temperatures in the presence of alkaline conditions (Zhu et al., 2020). The production of salts arises from the neutralization of these alkalines. These salts must be eliminated, which consumes tons of water and energy, for subsequent processes to continue. However, these extreme conditions affect the activity, and the stability of most known enzymes could be seriously affected (Sweeney and Xu, 2012). Therefore, enzymes that are stable in these extreme reaction conditions and in the presence of salts or tolerant to them are in great demand during downstream processes. Thus, the halo- and alkali-stable cellulases and hemicellulases identified in this study have the potential to hydrolyze lignocellulosic biomass for different industrial applications efficiently. In addition, the halotolerant nature of these enzymes offers an additional advantage because of their resistance to inactivation by salt residues that may result from pretreatment procedures (Zhang et al., 2011).

Furthermore, the biochemical characterization of the studied pectinase also showed high performance under alkaline, thermophilic, and halophilic conditions. There are several reports on alkaline and thermostable pectinase enzymes from various geographic locations and sources, particularly from agro- and industrial-wastes (Khatri et al., 2015; Oumer and Abate, 2017; Yu et al., 2017; Thakur et al., 2021). Regarding soda lakes, only two occurrences of pectin breakdown have thus far been described, both of which describe unique species of the phylum *Bacteroidota* belonging to the genera *Alkaliflexus* and *Natronoflexus* (Sorokin et al., 2014). At the same time, these microorganisms are low-salt tolerant saccharolytic fermentative alkaliphiles capable of hydrolyzing pectin. Thus, the poly-extreme nature of the pectinases in this study would make them ideal candidates for various industrial applications, particularly biomass degradation, retting and degumming of plant fibers, coffee and tea fermentation, and bio-scouring of cotton, all of which require extremes of temperature, pH, and ionic strength.

Halotolerant α-amylases have received considerably less consideration. However, most are thermostable and generate oligosaccharides in low-water or nonaqueous solvents, where hydrolytic processes are blocked (Masasa et al., 2022). This study’s amylases had optimum activity in the alkalinity range and at high temperatures. In addition, salinity enhanced the amylase activity of some clones, suggesting that these amylases are poly-extremophilic. Previously, haloalkaliphilic amylases were reported from various environments (Chakraborty et al., 2011; Moshfegh et al., 2013), including soda lakes in Ethiopia (Martins et al., 2001); however, the vast majority of these studies were from bacterial origins. Polyextremophilic α-amylase are extremely useful for the textile, food, brewing, and distilling industries, as has been argued in previous publications (Patel and Saraf, 2015).

In this study, most of the GH genes identified were found to likely originate from bacteria belonging to the genus of *Halomonas,* which corresponds to *Halomonas* as one of the most abundant genera in our previous work on the microbial diversity of Ethiopian lakes using amplicon metagenomics (Jeilu et al., 2022). *Halomonas* have, in previous investigations, been reported to be one of the most abundant prokaryotes in soda lakes (Humayoun et al., 2003; Dimitriu et al., 2008; Asao et al., 2011; Lanzén et al., 2013; Sorokin et al., 2014), responsible for the biogeochemical cycling and the immediate degradation of organic matter produced by autotrophic bacteria like *cyanobacteria* (Jones and Grant, 1999; Sorokin et al., 2014). Thus, we anticipate that *Halomonas* would generate a variety of hydrolytic enzymes, such as those necessary for the hydrolysis of complex polysaccharides, including cellulose, hemicellulose, pectin, and starch, which has also been indicated in a previous study (Tahrioui et al., 2013).

This study identified novel halo-alkaline and thermostable carbohydrate-degrading enzymes with potential applications in lignocellulosic biomass degradation. In order to use these enzymes in biofuel industrial applications, additional extensive molecular cloning, purification, and characterization studies are needed.

## Conclusion

Enzymes from halophiles and alkaliphiles are the most promising for biofuel generation and other industrial processes due to their inherent salt tolerance and thermal and alkaline stability. Soda lakes, in this aspect, are a one-of-a-kind source of extremophiles capable of harboring enzymes (extremozymes) that are active at both high pH and salinity. However, there is a scare of reports on searching CAZymes using culture-independent approaches from soda lakes. The present study has revealed the potential of functional metagenomics for exploiting the abundant genetic resources in uncultured microorganisms from extreme environments. Moreover, this study identified multiple families of GHs, indicating that the Ethiopian soda lakes constitute a unique biological niche for identifying novel CAZymes for applications in complete lignocellulose biomass degradations. Furthermore, many reported GH enzymes originated from mesophilic microorganisms where optimal activity and stability were around neutral pH and in the absence of salt. However, the biochemical characterization of the amylase, pectinase, and cellulase enzymes in this work shows that these enzymes are halo-alkaline and thermally stable. These properties strongly indicate the enzymes’ potential for use in various industrial processes, particularly biorefinery for lignocellulose biomass conversion.

## Data availability statement

The data presented in the study are deposited in the NCBI repository, accession number PRJNA799030. This data can be found at: https://www.ncbi.nlm.nih.gov/sra/PRJNA799030.

## Author contributions

OJ, AS, EA, EJ, and AG: designed the experiment and contributed to the study's conception and design. OJ: data collection and analysis, wrote the first draft of the manuscript. All authors contributed to the article and approved the submitted version.

## Funding

This study was financed by the Swedish International Development Cooperation Agency (SIDA) through the research and training grant awarded to Addis Ababa University and the Swedish University of Agricultural Sciences (AAU-SLU Biotech; https://sida.aau.edu.et/index.php/biotechnology-phdprogram).

## Conflict of interest

The authors declare that the research was conducted in the absence of any commercial or financial relationships that could be construed as a potential conflict of interest.

## Publisher’s note

All claims expressed in this article are solely those of the authors and do not necessarily represent those of their affiliated organizations, or those of the publisher, the editors and the reviewers. Any product that may be evaluated in this article, or claim that may be made by its manufacturer, is not guaranteed or endorsed by the publisher.

## Footnotes

^1^ https://github.com/gvarmaslu/WGS-fosmid-project

^2^ http://transdecoder.github.io
