# Supplementary figures and images for: Discovery of novel carbohydrate degrading enzymes from soda lakes through functional metagenomics
Source: Front Microbiol. 2022 Dec 7;13:1059061. doi: 10.3389/fmicb.2022.1059061 (PMC9768486; doi:10.3389/fmicb.2022.1059061)

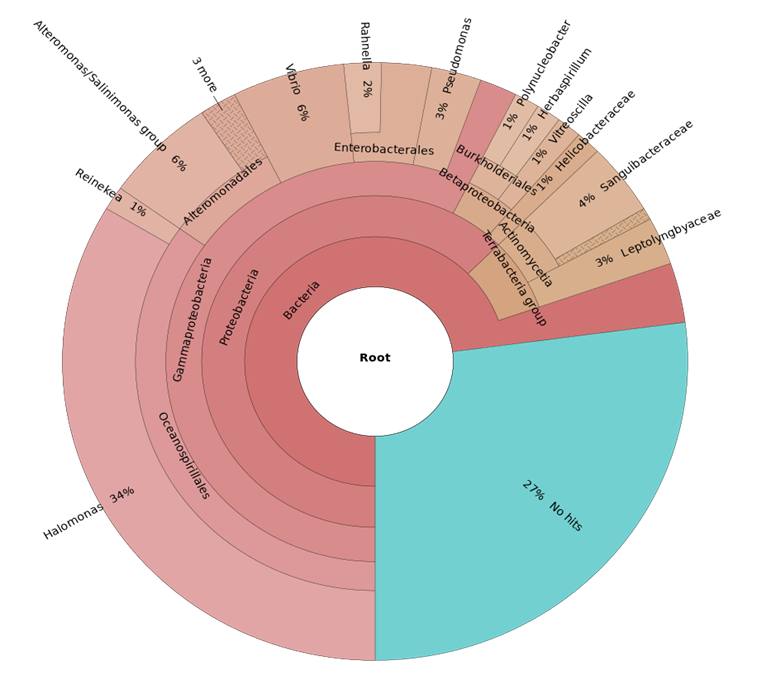

Supplement: Supplementary file 2 [file Image_1.TIF]
